# Interaction of Ge(Si) Self-Assembled Nanoislands with Different Modes of Two-Dimensional Photonic Crystal

**DOI:** 10.3390/nano12152687

**Published:** 2022-08-05

**Authors:** Margarita V. Stepikhova, Sergey A. Dyakov, Artem V. Peretokin, Mikhail V. Shaleev, Ekaterina E. Rodyakina, Alexey V. Novikov

**Affiliations:** 1 Institute for Physics of Microstructures Russian Academy of Sciences, 603950 Nizhny Novgorod, Russia; 2 Skolkovo Institute of Science and Technology, 143026 Moscow, Russia; 3 Radiophysical Department, Lobachevsky State University of Nizhny Novgorod, 603950 Nizhny Novgorod, Russia; 4 Rzhanov Institute of Semiconductor Physics, Siberian Branch of Russian Academy of Sciences, 630090 Novosibirsk, Russia; 5 Physical Department, Novosibirsk State University, 630090 Novosibirsk, Russia

**Keywords:** Ge(Si) self-assembled nanoislands, photonic crystal slab, microphotoluminescence, photonic crystal modes, bound states in the continuum

## Abstract

The interaction of Ge(Si)/SOI self-assembled nanoislands with modes of photonic crystal slabs (PCS) with a hexagonal lattice is studied in detail. Appropriate selection of the PCS parameters and conditions for collecting the photoluminescence (PL) signal allowed to distinguish the PCS modes of different physical nature, particularly the radiative modes and modes associated to the bound states in the continuum (BIC). It is shown that the radiative modes with relatively low Q-factors could provide a increase greater than an order of magnitude in the integrated PL intensity in the wavelength range of 1.3–1.55 µm compared to the area outside of PCS at room temperature. At the same time, the interaction of Ge(Si) islands emission with the BIC-related modes provides the peak PL intensity increase of more than two orders of magnitude. The experimentally measured Q-factor of the PL line associated with the symmetry-protected BIC mode reaches the value of 2600.

## 1. Introduction

It is believed that the problem of exponential growth of information volumes can be solved by the alliance of high-speed optical channels for its transmission with an integrated semiconductor technology for its processing [1,2,3]. The fabrication of compact, efficient light sources is one of the main problems here. In recent years, various micro- and nanoresonators, such as 1D (nanobeam) [4] and 2D photonic crystals with microcavity [5,6], plasmonic resonators [7,8], as well as single [9,10] and ensembles of Mie resonators [11], have been actively used to solve this problem. Significant progress in this area can be noted, the description of which can be found in the relevant reviews [12,13,14,15,16].

However, most of these studies were performed on A3B5 direct-gap materials, for which the integration issues with modern silicon CMOS technology, despite the impressive progress in recent years, remains unresolved [17,18]. This problem is much less acute for light-emitting structures based on Si and Ge, since SiGe heterostructures are already a part of the silicon technology (see, e.g., [19] and references therein). The use of microresonators makes it possible to significantly increase the efficiency of light sources based on these indirect-gap semiconductors [20,21,22,23,24] and achieve stimulated emission at low temperatures [25,26,27].

Among the various modes realized in dielectric resonators, the modes referred to as “bound states in the continuum” have recently attracted considerable interest [28,29,30,31,32,33,34,35]. The use of these states in microresonators based on direct-gap materials results in formation of high power [29], compact [32] and low threshold [35] lasers. Bound states in the continuum in SiGe-based heterostructures were experimentally observed in structures with Ge(Si) self-assembled nanoislands embedded in 2D photonic crystals [36,37,38]. Among various light-emitting SiGe heterostructures, the Ge(Si) islands have several properties that make them a convenient object for studying the interaction of an active medium with modes of dielectric resonators, including microcavities in photonic crystals [39,40,41] and Mie-resonators [42,43]. These structures have a luminescence signal at room temperature in the practically important spectral range of 1.3–1.55 μm [44,45]. Moreover, the spatial localization of charge carriers in the islands makes their light-emitting properties robust to the influence of various defects [46]. In addition, structures with Ge(Si) islands are characterized by a relatively simple formation compared to other light-emitting structures on silicon [47]. They do not need thick buffer layers [46] and can be grown directly on “silicon on insulator” (SOI) substrates, which provide a light confinement in the growth direction. Efficient interaction with the modes of dielectric resonators is also promoted by the high refracting index of silicon and germanium in the near-IR range [48].

In recent work, the authors demonstrated that the interaction of Ge(Si) islands with different modes of two-dimensional photonic crystal slabs (PCS) with a hexagonal lattice makes it possible to increase the intensity of their room temperature luminescence signal in the spectral region of 1.3–1.55 μm by more than two orders of magnitude [38]. Theoretical calculations have shown that due to the existence of degenerate doublet BIC modes at Γ-point, the contribution of BIC modes in the total modes number is higher in a PCS with hexagonal lattice than in a PCS with a square lattice [38]. This can lead to the active medium interactions exclusively with the BIC modes. However, the PCS parameters used in [38] did not allow to confidently identify the interaction of Ge(Si) islands with individual modes of different physical nature. In this work, the role of BIC modes in the luminescent response of Ge(Si) islands is analyzed in more detail in PCS with hexagonal lattice, exploiting a more careful choice of parameters of the initial structure and PCS itself. The specific behavior of these modes compared to radiative ones in a set of PCS with a wide range of parameters is discussed.

## 2. Materials and Methods

PCSs with hexagonal lattice were fabricated using electron beam lithography and plasma-chemical etching on multilayer structure containing five layers of self-assembled Ge(Si) nanoislands. The structure was grown by molecular beam epitaxy on a SOI substrate with a 3 μm thick buried oxide layer and a Si device layer thinned down to 90 nm. The structure were grown at the temperature of ~600 °C because dome-shaped Ge(Si) islands formed at this temperature demonstrate the highest emission intensity at room temperature [45]. The grown structure consisted of a 40 nm thick Si buffer layer, 5 layers of Ge nanoislands separated by 12 nm thick Si spacer layers, and a 40 nm thick Si cap layer. The total thickness of the structure above the buried oxide was 220 nm, which is less than the thickness of structure (300 nm) analyzed in [38]. Reducing the total thickness of the structure made it possible to etch PCS over the entire thickness of structure down to the buried oxide using plasma-chemical etching in a mixture of SF_6_/C_4_F_8_ gases and a PMMA resist as a mask. Additionally, according to the calculations of the emissivity dispersion performed during rigorous coupled-wave analysis (RCWA) [49], the small thickness of the structure ensured a sufficiently large spectral distance between different PCS modes, which makes it possible to establish a direct relationship between the individual lines observed in the PL spectra and the selected PCS modes.

**Figure 1:**
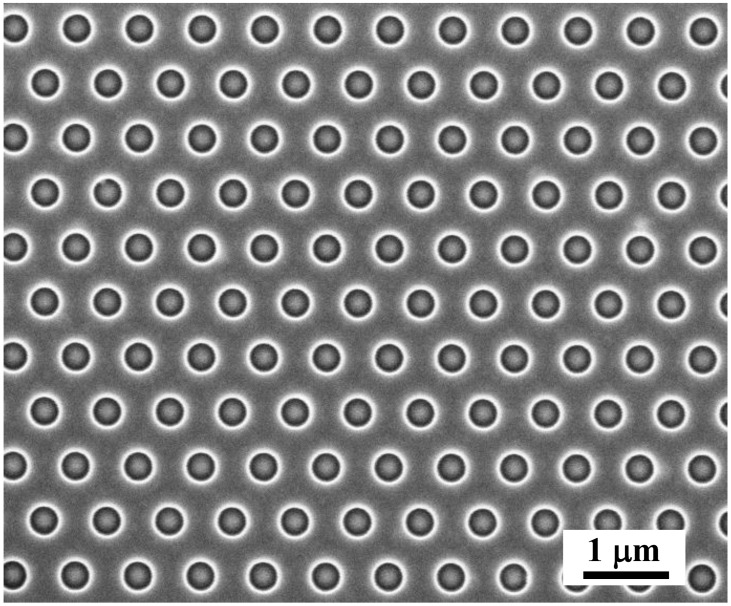
The SEM image of a part of the PCS with *a* = 700 nm and *r*/*a* = 0.25.

The lattice period (*a*) of the fabricated PCSs varied in the range of 525–1000 nm. The hole radius (*r*) to period ratio (*r*/*a*) and the number of PCS periods were fixed at ~0.25 and 35, respectively, for all studied PCSs. A typical scanning electron microscopy (SEM) image obtained for investigated PCSs is shown in Figure 1.

**Figure 2:**
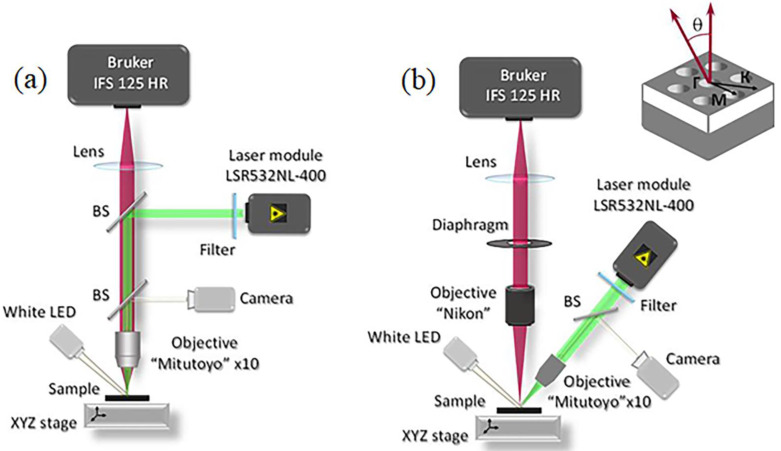
Schematics view of (**a**) the micro-photoluminescence (micro-PL) and (**b**) the directional photoluminescence (DPL) setups. BS in the figures—beam splitters. A hexagonal photonic crystal, it’s the high symmetry directions and the signal collection angle Θ are schematically shown in the upper right corner.

Experimental studies of the luminescence properties of the fabricated PCSs were carried out using two micro-photoluminescence setups, described in detail in [38] and schematically presented on Figure 2. In the first one, which was the standard micro-PL setup, the PL signal was excited and collected using the same objective located normal to the sample surface (Figure 2a). In the micro-PL setup, the spatial resolution and the fixed signal collection angle are determined by the objective. For the used «Mitutoyo» M Plan APONIR 10× objective with numerical aperture NA = 0.26, the size of the region under study was 10 μm, which is smaller than the PCS size, and the PL signal was collected in a solid angle 2Θ ~ 30°.

In the 2-nd scheme, namely the so-called “directional photoluminescence” (DPL) setup, different objectives were used for excitation and collection of the PL signal (Figure 2b). In the DPL setup, the same Mitutoyo M Plan APONIR 10x objective was used for excitation of the PL signal, but it was located at an angle of ~60° with respect to the surface normal. The second objective («Nikon» 50 mm f/1.4D AF Nikkor, NA = 0.37) was located at the focal distance from the sample surface. In such geometry, the emitting area can be considered as a point source. Accordingly, the light beam formed by this objective can be regarded as parallel. A diaphragm located in this parallel beam, whose diameter can be varied over a wide range, made it possible to controllably change the PL collection angle. The collection angle varied from a few degrees to a value of 2Θ ~ 44°, which is determined by the numerical aperture of the objective.

In both PL setups, the luminescence signal was excited by a solid-state CW laser emitting at the wavelength of 532 nm (laser module LSR532NL-400) (Figure 2). To detect the signal, a high-resolution Fourier spectrometer (Bruker IFS 125 HR) (Figure 2) and a nitrogen-cooled Ge photodetector were used. The spectral resolution in both experimental schemes could reach 0.05 cm^−1^. All PL measurements were carried out at room temperature.

## 3. Results and Discussion

**Figure 3:**
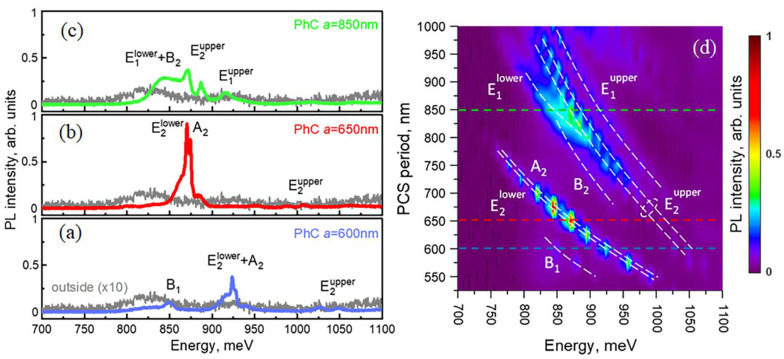
(**a**–**c**)—spectra measured using micro-PL setup in the area outside of the PCSs and within the PCSs with periods *a* = 600 nm (**a**), 650 nm (**b**) and 850 nm (**c**) The spectrum measured in the area outside the PCSs is multiplied by 10. (**d**)—2D representation of micro-PL spectra as a function of energy and PCS period. The dashed lines of the corresponding color denote the spectra of PCSs presented in figures (**a**–**c**). The letters denote the PCS’s modes associated with the corresponding peaks in the micro-PL spectra (modes designation taken from [38]).

In the PL spectra measured using the micro-PL setup, the as-grown sample demonstrated relatively weak signals related to Si band edge transitions in the energy range of 1050 ÷ 1150 meV as well as signals related to the different optical transitions in Ge(Si) nanoislands in the energy range of 750 ÷ 950 meV (Figure 3a) [44,45,50]. The shape of these signals from the as-grown sample is significantly affected by the interference effects caused by multireflection from the buried oxide layer of the SOI substrate. The formation of a PCS periodic structure leads to the appearance of individual lines of various widths in the micro-PL spectra. Peak intensity of these lines significantly exceeds the intensity of PL signal from the unprocessed sample area (Figure 3a–c). These lines are associated with the interaction of Ge(Si) nanoislands with photonic crystal modes of different physical nature located above the light cone [38].

**Figure 4:**
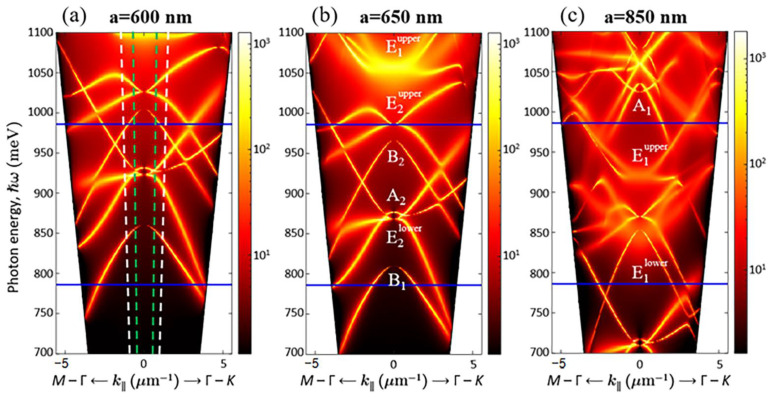
The emissivity dispersion dependences of PCS modes calculated for PCS with the periods *a* = 600 nm (**a**), 650 nm (**b**) and 850 nm (**c**). The dashed lines in figure (**a**) limit the signal collection angle in the micro-PL setup (white lines, 2θ ~ 30°) and in the DPL setup for a diaphragm diameter of 10 mm (green lines, 2θ ~ 15°). The first-order quasi-guided modes are marked in figures (**b**,**c**) according to [38]. Horizontal blue lines mark the energy range of PL signal related with Ge(Si) nanoislands.

As shown by theoretical analysis using the group theory, the number of the first-order quasi-guided modes in the PCS with hexagonal lattice is 12 [38]: four singlet modes (A_1_, A_2_, B_1_ and B_2_) and eight doublet modes (E_1_, E^1^, E_2_ and E^2^) degenerated in the Γ-point of the Brillouin zone (Figure 4) (modes designation corresponds to irreducible representations of C_6v_ group). The superscripts “lower” and “upper” show the relative energy position of the modes of the same symmetry. Among these 12 modes, only E_1_ and E^1^ doublet modes are the radiative low-Q modes. The remaining eight modes are characterized by a high Q factor in the Γ-point (Figure 4) that is attributed to their BIC origin [38]. As mentioned in Section 1, great interest in these states is due to their extremely high Q factors, despite the fact that they are located in the continuum of states above the light cone [27,28,29,30,31,32,33,34,35,36,37,38].

As mentioned above, a standard micro-PL setup collects a signal that falls within a fixed solid angle relative to the normal to the sample surface (Figure 2a). This angle is determined by the objective’s numerical aperture and equals to 2Θ ~ 30° (marked by white dashed lines in Figure 4a). On the one hand, a large collection angle increases the intensity of the detected PL signal and makes it possible to detect PCS modes with low emissivity near the Γ-point such as B_1_ and B_2_ modes (Figure 3 and Figure 4). At the same time, a non-flat dispersion of the PCS modes leads to broadening of individual PL lines and their spectral overlapping with other modes (Figure 3). Such a spectral overlap is observed, for instance, for closely spaced E_2_ and A_2_ modes (Figure 3a,b). This circumstance complicates the interpretation of the spectra noticeably.

**Figure 5:**
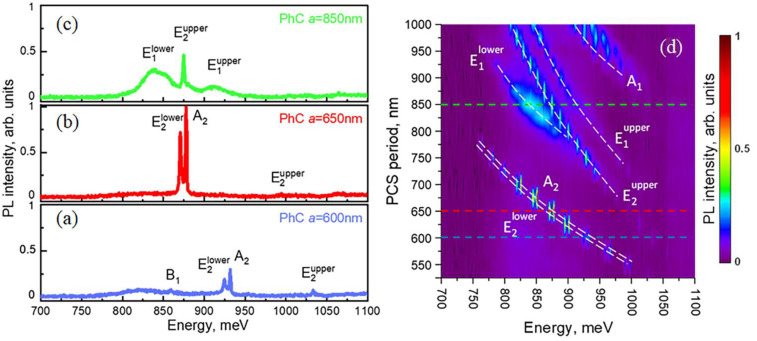
(**a**–**c**)—The spectra measured using the DPL setup with 2Θ ~ 15° for PCSs with *a* = 600 nm (**a**), 650 nm (**b**) and 850 nm (**c**). (**d**)—2D representation of DPL spectra as a function of energy and period of measured PCSs. The dashed lines of the corresponding color denote spectra of PCSs presented in figures (**a**–**c**). The letters denote the PCS’s first order modes associated with the corresponding peaks in the DPL spectra (see also Figure 3). The unmarked luminescence signal in figure (**d**) in the energy range ≥ 950 meV for PCSs with *a* ≥ 900 nm is associated with the PCS’s second order modes.

The DPL setup described above makes it possible to control the PL collection angles (2Θ) by changing a diameter of the diaphragm placed in a parallel beam formed by the collecting objective (Figure 2b). While choosing the optimal collection angle for PL measurements, one should keep in mind that the smaller 2Θ the lower the detected PL intensity, but at the same time, the better angular resolution one can obtain. Thus, by reducing the collection angle in the DPL setup by half in comparison with the micro-PL setup (from 2Θ ~ 30° to 2Θ ~ 15° (Figure 4a)), it appears to be possible to spectrally separate the PL lines associated with E_2_ and A_2_ modes (Figure 5a,b). In addition, a decrease of the collection angle results in the observation of E^2^ doublet in the form of only one narrow line (Figure 5c) in contrast to the case of using the micro-PL setup where the same mode was seen as a double peak resonance in the PL spectra. As a result, The DPL spectra measured with 2Θ ~ 15° allow to establish the unambiguous relationship between the PL lines and PCS modes (Figure 4 and Figure 5). In this case, the spectral coincidence of the experimentally observed PL lines with their theoretically calculated positions at the Γ point is ±10 meV.

However, due to a small collection angle in the DPL setup, the PL signal from the unprocessed area for 2Θ ≤ 15° has a very low signal-to-noise ratio. Moreover, at these measurement conditions, the intensity of the PL signal associated with B_1_ and B_2_ BIC modes is also very low. A rapid decrease of the PL signal associated with these modes with decreasing collection angle is explained by a wider range of *k* values near the Γ-point of the Brillouin zone (in comparison with other BIC modes), where their emissivity in the far field is low (Figure 4). Theoretical simulations predict a wide range of the wave-vectors near the Γ-point, where these modes have low emissivity and high Q-factors, though it should depend on the losses in real PCSs [38].

The differences in the intensity and the linewidths in the DPL spectra clearly demonstrate the different physical nature of various PCS modes. For example, radiative doublets E_1_ are characterized by a low Q factor and large PL linewidth (Figure 5c). Despite the relatively low peak intensity, the lines associated with these modes provide a significant increase in the integrated intensity of the PL signal from Ge(Si) nanoislands. As a result, the maximum enhancement of the integrated PL intensity from nanoislands in PCSs relative to the unprocessed area is achieved for the PCS with period *a* = 850 nm. For this period, two radiative doublet modes E_1_ fall into the energy range of nanoislands emission (Figure 3 and Figure 5). The enhancement factor exceeds one order of magnitude for the micro-PL spectra. In the case of DPL spectra, it is difficult to evaluate the enhancement factor because a small collection angle (2Θ < 15°) provides too low PL intensity from the unprocessed area.

**Figure 6:**
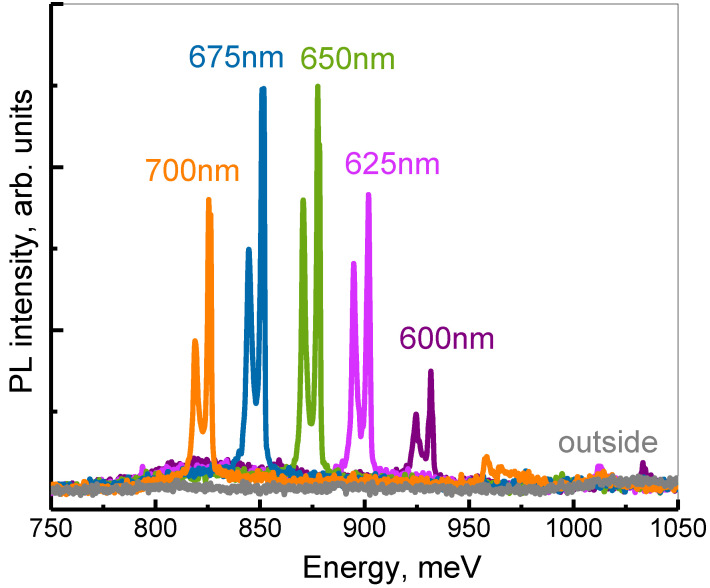
The DPL spectra of the PCSs with different periods (*a* = 600 ÷ 700 nm) in comparison to DPL spectra of the area outside of PCS. The collection angle is 2Θ ~ 15°.

At the same time, due to high Q-factors, all of the PL lines associated with the BIC modes have small linewidths and high peak intensity (Figure 5). As was noted above, the highest absolute PL intensity in the studied PCS has been determined for the spectrally close BIC modes E_2_ and A_2_ (Figure 5). This is due to their high emissivity near the Γ-point [38] and also due to a relatively large spectral distance from other PCS modes. The former is due to the small thickness of the structure above the buried oxide (Figure 4). As a result, only two lines associated with these BIC modes are observed in the spectral range of Ge(Si) nanoislands emission in DPL spectra measured for PCSs with periods *a* = 600 ÷ 700 nm (Figure 6). Interaction of Ge(Si) nanoislands with these modes makes it possible to significantly increase the PL intensity at room temperature in the practically important spectral wavelength range of 1.3–1.5 μm (Figure 6). The largest enhancement of the PL intensity is observed for PCSs with periods a = 650 ÷ 675 nm, when the energy position of the E_2_ and A_2_ modes coincides with the maximum of Ge(Si) nanoislands luminescent response in the as-grown structure. In the PL spectra measured by the micro-PL setup, the increase in the peak PL intensity from nanoislands reaches ~110 times. As in the case of the radiative modes described above, the PL intensity enhancement on BIC modes is difficult to assess correctly from the DPL spectra because of the low intensity of the PL signal measured from the unprocessed area (Figure 6).

**Figure 7:**
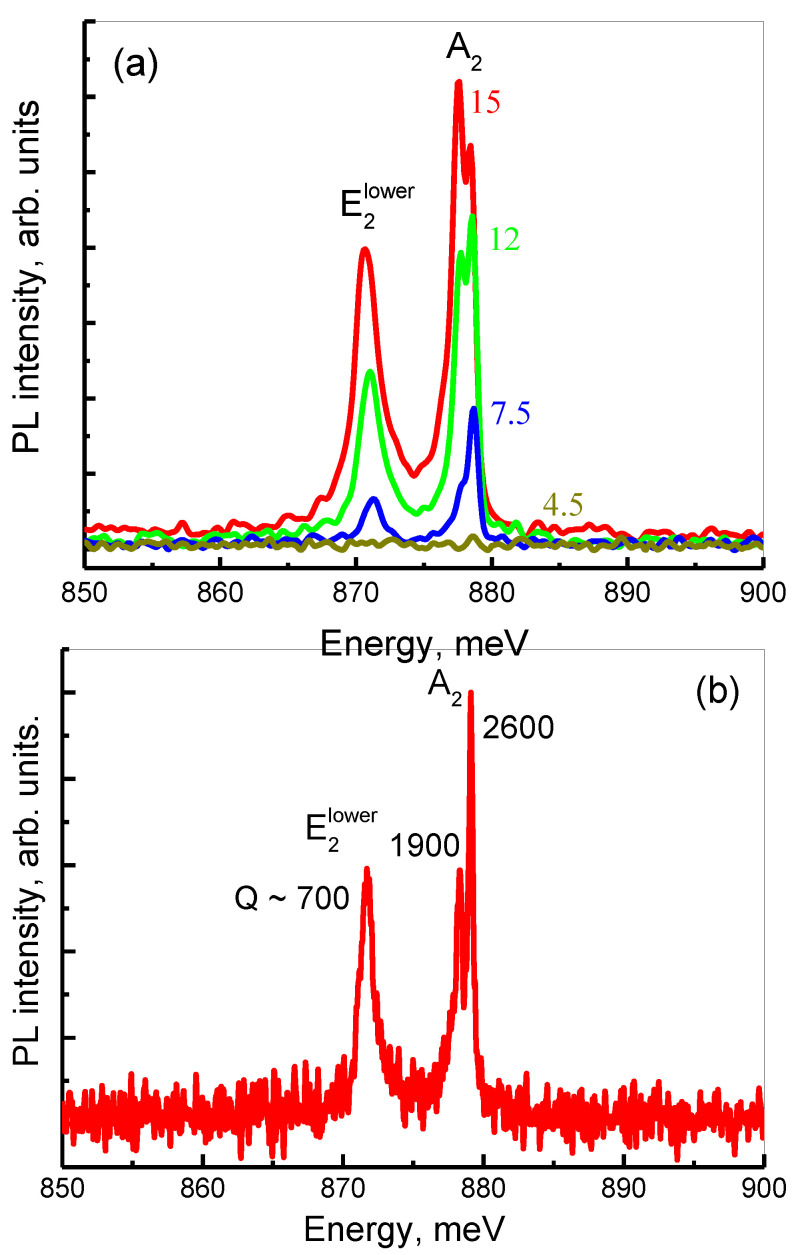
(**a**) The DPL spectra of PCS with *a* = 650 nm measured in the spectral range of PL lines associated with E_2_ and A_2_ modes. The spectra were measured within various collection angles with the spectral resolution of 4 cm^−1^. The signal collection angles (2Θ) are indicated next to the corresponding spectrum. (**b**)—DPL spectrum of PCS with *a* = 650 nm measured within collection 2Θ ~ 6° with the spectral resolution of 0.5 cm^−1^. The Q factors values are indicated next to the observed PL lines.

It is known that the quality factor of symmetry-protected BIC modes for PCSs increases sharply as the Γ-point is approached [32,38]. In this regard it is important to analyze the change in the width of the PL lines associated with BIC modes as the collection angle decreases. Such an analysis was carried out for a PCS with *a* = 650 nm for which the most intense PL lines associated with the A_2_ singlet and E_2_ doublet BIC modes are observed (Figure 7). One can see a decrease of the PL intensity of these modes with the decrease of the collection angle (Figure 7a). The minimum collection angle at which it is possible to detect a PL signal from A_2_ singlet and E_2_ doublet BIC modes is 2Θ ~ 5° (Figure 7a). With the decrease of the collection angle, there is no noticeable change of the linewidth and the Q-factor of the doublet E_2_ BIC mode. Indeed, the Q-factor of this mode, determined from the PL linewidth measured with a spectral resolution of 4 cm^–1^, stays in the range of Q ~ 600 ÷ 700. The relatively low experimentally determined Q factor of the doublet BIC mode is explained by the lifting of its degeneracy at *k* ≠ 0 and the different dispersion dependences of the components of this mode (Figure 4), which leads to the broadening of the PL line.

**Figure 8:**
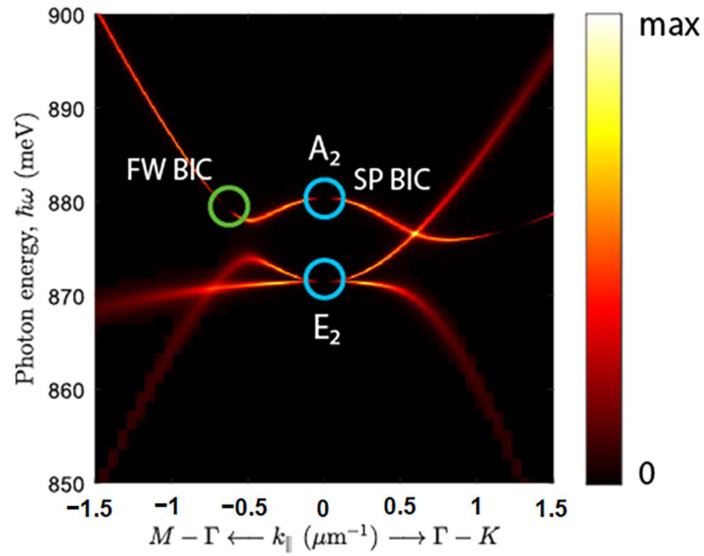
Calculated emissivity near modes A_2_ and E_2_. Blue and green circles denote symmetry-protected (SP) and Fridrich-Wintgen (FW) BIC modes, respectively. Linear color scale is shown on the right.

An analysis of the PL line shape of the A_2_ singlet measured with a small signal collection angle shows that this line has a fine structure consisting of two peaks (Figure 7a). It can be seen that the intensity of the lower-energy peak in these spectra decreases faster with the decrement of the collection angle than that of the high-energy peak (Figure 7a). At the smallest collection angle (2Θ ~ 6°), these two peaks can be resolved spectrally by increasing the spectral resolution of the spectrometer from 4 cm^−1^ to 0.5 cm^−1^ (Figure 7b). The low-energy peak observed in the signal associated with the A_2_ mode can be related with the presence of the so-called accidental or Friedrich–Wintgen (FW) BIC state [38]. The FW-BIC is a result of destructive interference of two closely spaced modes which in PCS with *a* = 650 nm is realized close to the Γ-point of the first Brillouin zone (Figure 8), [38,51,52]. In the studied PCS, both the FW BIC and SP BIC are located in the close energy range. For this reason, these two types of BIC are difficult to distinguish in the experimentally measured PL spectra.

The BIC nature of the two peaks observed at the photon energies close to theoretical A_2_ mode photon energy is confirmed by their small linewidth and, consequently, by their high Q-factors. The Q factors determined for these peaks from the analysis of DPL spectrum measured within 2Θ ~ 6° and the spectral resolution of 0.5 cm^−1^ are: Q ~ 1900 for the low-energy peak associated with the FW BIC and Q ~ 2600 for the high-energy peak associated with the symmetry protected BIC (Figure 7b). These values are much higher than the Q-factor of the peak associated with the doublet E_2_ mode (Figure 7b). It is noteworthy that the experimental quality factor Q ~ 2600 determined for the singlet A_2_ exceeds the values demonstrated in our previous work [38]. Moreover, this experimentally obtained Q factor is comparable with those determined for BICs in the lasing regime in direct-gap semiconductors [53,54] and concedes only with the Q ~ 7300 achieved in Ref. [35], where the lasing of the so-called super-BIC was reported.

## 4. Conclusions

In conclusion, the interaction of Ge(Si)/SOI self-assembled nanoislands with PCS modes of different physical nature (radiative and bound states in the continuum (BIC) modes) has been analyzed separately with the help of the proper selection of the PL measurements conditions and the PCS parameters (thickness, period and hole radius-to-period ratio). It has been shown that, depending on the type of the PCS mode, it is possible to achieve either a significant increase in the integrated PL intensity (by more than an order of magnitude) or the peak PL intensity (by more than two orders of magnitude) in the wavelength range of 1.3–1.55 µm at room temperature. The experimentally measured quality factor for the PL line associated with the symmetry protected BIC mode reaches the value of 2600. The obtained results can be used for the development of compact high-efficiency light sources for silicon photonics.

## Data Availability

Not applicable.
